# Gamma-ray spectra and absorbed doses measured at EuXFEL undulator system

**DOI:** 10.1107/S1600577525006605

**Published:** 2025-08-27

**Authors:** Olga Falowska-Pietrzak, Anders Hedqvist, Fredrik Hellberg, Frederik Wolff-Fabris, Niels Bassler

**Affiliations:** a Department of Physics Stockholm University Stockholm Sweden; b European XFEL Holzkoppel 4 22869Schenefeld Germany; c Department of Clinical Medicine Aarhus University Aarhus Denmark; d Danish Centre for Particle Therapy Aarhus University Hospital Aarhus Denmark; Advanced Photon Source, USA

**Keywords:** EuXFEL, undulators, *Geant4*, gamma-ray spectroscopy, RADFET

## Abstract

Characterization of the stray radiation field present in the undulator systems at the European XFEL GmbH (EuXFEL) is of great importance, as the potential damage to undulator permanent magnets, electronics and diagnostic equipment depends both on the type of particles and the energy. This work presents the energy profile of the stray radiation measured in the upstream and downstream part of the undulator system near the beam pipe. The influence of machine operation settings on the radiation field intensity was also investigated. A comparison between gamma-ray energy measurements (30 keV to 1.5 MeV) and *Geant4* Monte Carlo simulations shows that stray radiation in the upstream part of the undulator system originates from high-energy electrons intersecting the beam pipe wall. The intensity of the measured signal is approximately linear to the charge passing through the undulator system and to the electron energy but is inversely proportional to the square root of the undulator gap. Measurements in the upstream part of the undulator system show that the stray radiation level can be mitigated with careful choice of accelerator settings. The intensity of the radiation with energies below approximately 400 keV is higher in the downstream part of the undulator system compared with the upstream part, during regular operation. A CZT spectrometer and RADFET measurements confirm that the radiation field in the downstream part of the undulator system is dominated by low-energy synchrotron radiation (below 200 keV), which constitutes approximately 99% of the stray radiation field.

## Abstract 2

The origin and composition of the stray radiation at the EuXFEL undulator system are investigated. This is essential for machine protection, as the stray radiation can damage the undulator permanent magnets as well as the correction and diagnostic equipment along the beamline.

## 1. Introduction

The European X-ray Free Electron Laser (EuXFEL) is a free-electron laser (FEL) facility producing ultrashort, coherent X-ray pulses through the self-amplified spontaneous emission (SASE) process (Kim, 1986 ▸). FEL radiation is generated as electrons, accelerated to energies up to 17.5 GeV in an almost 2 km long supercoducting linear accelerator, travelling through long periodic arrangements of Nd_2_Fe_14_B permanent magnets, called undulators (Altarelli *et al.*, 2006 ▸). EuXFEL is a high duty cycle linear accelerator where electrons are injected at a maximum repetition rate of 27000 bunches per second with bunch charges up to 1 nC. Measurements of absorbed doses near the undulators indicate the presence of stray radiation outside the beam pipe. It is potentially damaging not only to the permanent magnets but also to diagnostic and correction equipment located along the beam pipe. Permanent magnet demagnetization at EuXFEL was reported earlier for a diagnostic undulator located directly upstream of the undulator system (Wolff-Fabris *et al.*, 2018 ▸). The magnetic degradation reached 3.5% after 4.4 kGy of absorbed dose. As the change in magnetic field alters the conditions for phase matching between the electron and the photon field, the accelerator performance may eventually be affected. The severity of the magnets’ damage depends both on the particle type and energy, which has been extensively summarized previously (Samin, 2018 ▸). It is then of great importance to characterize the stray radiation field near undulators in these terms to be able to mitigate its effects.

**Figure 1:**
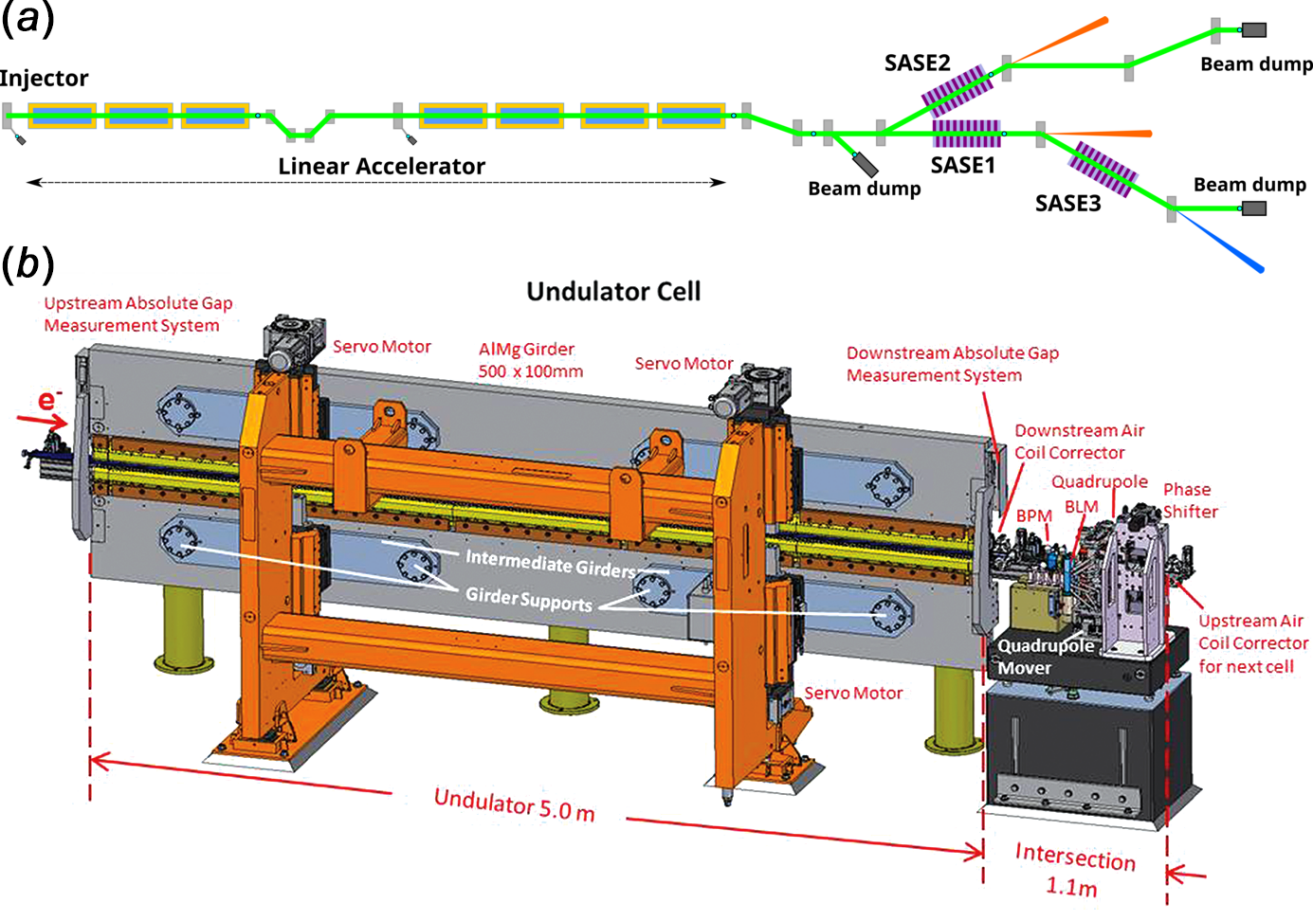
Schematic layout of the EuXFEL facility. (*a*) Electrons emitted from the injector are accelerated to energies up to 17.5 GeV in the linear accelerator. Electron bunches are then distributed to three undulator systems: SASE1, SASE2 and SASE3. (*b*) Undulator cell of the undulator systems of the EuXFEL (Abeghyan *et al.*, 2019 ▸).

The layout of EuXFEL is presented schematically in Fig. 1 ▸(*a*). X-ray pulses are created in one of three undulator systems: SASE1, SASE2 and SASE3, with SASE3 located directly downstream of SASE1. Electron bunches accelerated in the linear accelerator are distributed to different undulator systems with kicker magnets which are a part of a beam distribution system. There is one common electron beam transport line for SASE1 and SASE3, which means that electron bunches directed towards SASE3 travel through the SASE1 line (Liu *et al.*, 2019*a* ▸).

The undulator system consists of a number of undulator cells, each in turn consisting of an undulator segment (5 m long) and an intersection (1.1 m long), as presented in Fig. 1 ▸(*b*). SASE1 and SASE2 have 35 undulator cells and produce FEL radiation with a wavelength between 0.05 nm and 0.4 nm (Abeghyan *et al.*, 2019 ▸). The SASE3 system contains 21 undulator cells and produces FEL radiation of wavelength between 0.4 nm and 5.2 nm. Undulator segments consist of permanent magnets mounted on two movable girders, below and above the beam pipe. The distance between the top and bottom permanent magnets is referred to as the ‘undulator gap’ (or simply ‘gap’). In the intersection, the correction and diagnostic equipment such as the quadrupole magnet, air coil correctors, permanent magnet phase shifter, beam position monitor and beam loss monitor are installed to correct the trajectory of the electron beam and to match the electron phase between adjacent undulator segments (Altarelli *et al.*, 2006 ▸).

The source of the stray radiation measured outside the beam pipe in the undulator beamlines is different in the upstream and downstream parts of undulator systems (Liu *et al.*, 2019*b* ▸). In the upstream undulator cells, the stray radiation field is created as high-energy electrons hit the beam pipe, either as a result of the misalignment of the main electron beam, the electron halo deviated from the main beam trajectory or a dark current coming from the upstream of the undulator system. The dark current is measured when electrons are not transported in the linac (beam off) and it is believed to be produced in the linear accelerator by field emission (Lipka *et al.*, 2011 ▸). The dark current is most likely present with beam on. The origin of the electron halo in the undulator systems is not fully understood. It is defined as low intensity electrons with extended spatial distribution compared with the main beam. This can be caused by, for example, non-linear beam dynamics, space-charge effects and electron beam scattering. At EuXFEL, electron halo measurements are performed with wire scanners (Liu *et al.*, 2017 ▸). They are installed upstream and downstream of the post-linac collimation system which collimates both dark current and electron halo. First halo measurements at EuXFEL showed that, after the collimation section, the electron halo at −10σ is reduced by at least one order of magnitude in the horizontal direction and two orders of magnitude in the vertical direction. In the downstream part of the undulator system, the stray radiation outside the beam pipe is dominated by the synchrotron radiation associated with the SASE process.

The absorbed dose at each EuXFEL undulator segment is continuously monitored by silicon-based radiation-sensitive p-MOSFET RADFET dosimeters (Schmidt-Foehre *et al.*, 2012 ▸). In addition, two LB 6419 area monitors register neutron and gamma dose rates in both upstream and downstream parts of SASE1 (Klett *et al.*, 2010 ▸). The spatial distribution of the absorbed dose along the undulator segments and in the intersections was previously measured with Gafchromic films (Falowska-Pietrzak *et al.*, 2022 ▸; Falowska-Pietrzak *et al.*, 2023 ▸). This present work aims to characterize the photon component of the stray radiation field in different parts of the undulator system with respect to its energy profile. For this, we analyse RADFET absorbed doses and gamma-ray energy spectra measurements performed in the upstream and downstream parts of the SASE1 undulator system during regular user operation and compare the results with numerical simulations. The impact of individual machine settings (*e.g.* electron beam energy and undulator gap) on the stray radiation field is also investigated.

## 2. Materials and methods

### 2.1. Experimental techniques

The photon energy distribution outside the beam pipe in two SASE1 undulator cells was measured with two Kromek GR05+ solid-state gamma-ray spectrometers (Kromek Ltd, https://www.kromek.com). These are semiconductor detectors that utilize a cadmium–zinc–telluride crystal (referred to later as CZT) as a sensitive volume. Compared with other commonly used semiconductor materials such as germanium and silicon, CZT-based crystals can be effectively used for room-temperature measurements due to their larger bandgap of 1.57 eV (Chakraborty & Hashmi, 2019 ▸). The high atomic numbers of the CZT constituents makes the detector more sensitive to high-energy photons.

The spectrometer’s sensitive volume of 0.125 cm^3^ is embedded in an aluminium casing (6.3 cm × 2.5 cm × 2.5 cm). The energy range of the spectrometer is between 30 keV and 3 MeV, with a maximum throughput of 50000 counts per second. The spectrometer’s resolution at 662 keV is less than 2% full width at half-maximum. Its small size makes it suitable for photon spectra measurements at places difficult to access for other detectors. The GR05+ spectrometer is directly connected to a computer with a mini-USB cable, acting also as a power supply.

The first spectrometer was installed in cell #19 in the SASE1 tunnel in 2023 to measure the low-energy radiation associated with the SASE process. The second spectrometer was first characterized at Stockholm University in 2023 in terms of energy, resolution and efficiency (Falowska-Pietrzak *et al.*, 2024 ▸). It was installed a few months later in SASE1 cell #3 to pick up possible radiation emitted as a result of electrons interacting with the beam pipe. Both of the spectrometers were placed close to the entrance of the undulator segment, at the height of the beam pipe but slightly off-axis (around 10 cm). Spectra were collected simultaneously in both cells for about one year of regular user operation at EuXFEL. The accelerator settings such as undulator gap or number of electron bunches passing through SASE1 might change frequently depending on the user requirements. To account for this, the measurement time for individual spectra was set to 10 min or less. The conditions during which the charge rate, electron energy and undulator gap are constant are later referred to as constant accelerator settings.

Radiation detection silicon-based sensors RADFETs are commonly used at particle accelerator facilities (Fröhlich *et al.*, 2013 ▸; Han *et al.*, 2018 ▸). At EuXFEL, among different purposes, they are employed to provide real-time measurements of the absorbed dose to the undulator segments, with a dose range varying between 0.1 Gy and 20 kGy. The RADFETs are mounted on the upstream side of every undulator segment, attached to undulator girders, above and below the beam pipe. In the configuration where the undulator gap is movable, the RADFET’s distance to the beam pipe in the vertical direction follows the position of the permanent magnets with respect to the beam pipe when operating at different photon energies. In 2018, all of the bottom RADFETs in the SASE1 undulator tunnel were encapsulated with a 4 mm lead shield. This suppresses gamma absorbed doses with energy lower than around 200 keV, based on the standard calculation of photon penetration in lead.

### 2.2. *Geant4* Monte Carlo simulations

Electron losses in the upstream part of the SASE1 undulator system were simulated using the *Geant4* simulation toolkit (Agostinelli *et al.*, 2003 ▸). The geometry defined in the simulation consisted of two 5 m-long undulator segments (with Nd_2_Fe_14_B magnets attached to aluminium support) separated by a 1.1 m-long intersection. Diagnostic and correction equipment present in the intersection at EuXFEL impacts the stray radiation present outside the beam pipe near the undulator magnets. Therefore, absorber, vacuum pump, beam position monitor, beam loss monitor, quadrupole magnet, phase shifter and a pair of air coils correctors were included in the simulations. The majority of the intersection components included in the code were made of aluminium. Other materials were iron in the quadrupole magnet, Nd_2_Fe_14_B magnets in the phase shifter and copper in the absorber design.

**Figure 2:**
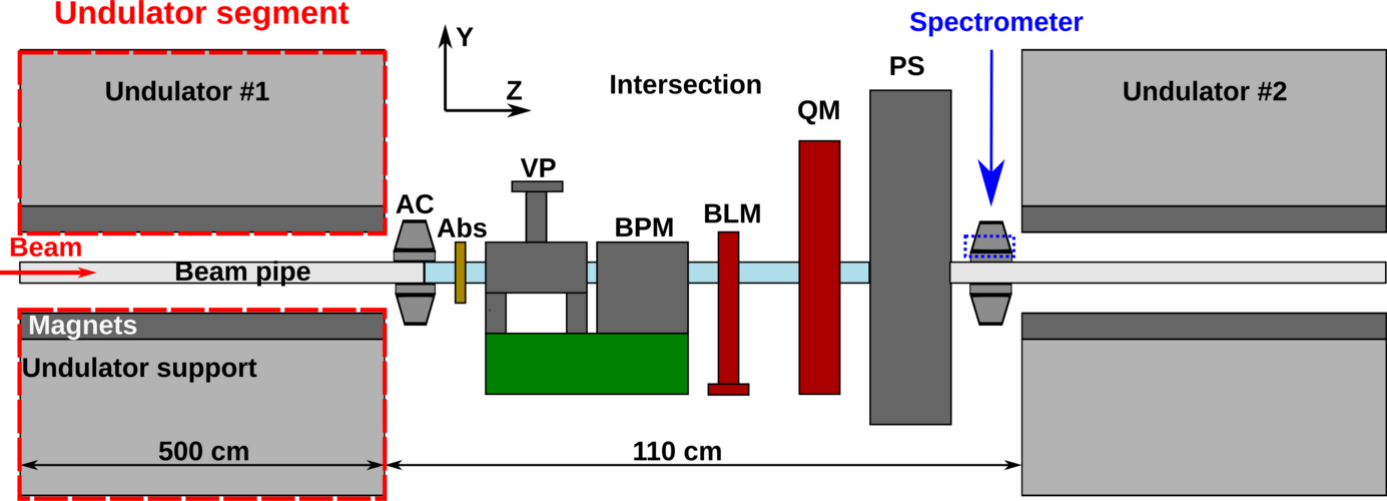
Schematic presentation of the geometry used in the simulations. AC: air coil; Abs: absorber; VP: vacuum pump; BPM: beam position monitor; BLM: beam loss monitor; QM: quadrupole magnet; PS: phase shifter. The CZT spectrometer is located behind the downstream air coil. The cylindrical beam pipe is marked with blue colour.

The CZT spectrometer was added upstream of the second undulator segment, at the height of the beam pipe. Schematic presentation of the described geometry is shown in Fig. 2 ▸.

At EuXFEL, the shape and aperture of the beam pipe is not constant along the undulator cell (Altarelli *et al.*, 2006 ▸) and was considered in the simulations. The aluminium beam pipe is rectangular (70 mm × 9.6 mm) in the undulator segments, with an elliptical aperture (diameter of 15 mm and 8.8 mm in both the horizontal and vertical directions). The beam pipe changes to cylindrical (with 0.5 mm thick wall and 9 mm diameter aperture) in the beginning of intersection, approximately at the position of the absorber. Throughout the simulations, the undulator gap was kept constant at 14 mm which corresponds to 9.6 keV FEL radiation for an electron beam at 14.0 GeV. The simulated electron beam energy was chosen to be 14.1 GeV because the actual electron energy, denoted as 14.0 GeV, can vary by a few MeVs due to the precision of the measurement.

A summary of simulation parameters is given in Table 1 ▸. For user operation at EuXFEL, selected electron beam energies of 11.5, 14.0 and 16.3 GeV are mostly used. As simulations were to replicate the electron beam halo, the source was defined as a ring of electrons. The size of the ring was chosen for the electrons to interact with the beam pipe at the point where its aperture changes – approximately at the position of the absorber. The particles were generated using the *G4GeneralParticleSource*, which in contrast to another particle generator available in *Geant4* called *G4ParticleGun* allows the shape of the source to be specified (Geant4 Collaboration, 2021*a* ▸). Electrons then interact with the beam pipe at the first point when the aperture changes from elliptical to cylindrical. *Geant4* physics reference list FTFP_BERT_HP, applicable for high-energy physics applications, was used in the simulations (Geant4 Collaboration, 2021*b* ▸). It consists of both hadronic and electromagnetic components, and includes the radioactive decay of unstable nuclei. The spectrum of the energy deposited inside a CZT crystal was registered as a simulation output.

## 3. Results and discussion

### 3.1. Upstream part of SASE1 – cell #3

**Figure 3:**
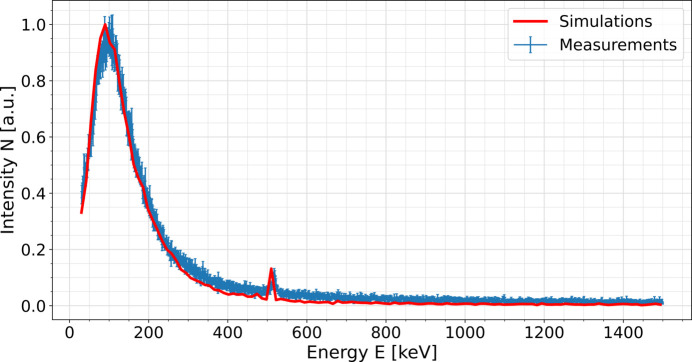
Comparison between measured (blue) and simulated (red) gamma-ray spectra. The data are a sum of 10 min measurements in a total measurement time of 80 min.

Fig. 3 ▸ shows a comparison between measured and simulated gamma-ray spectra. The spectrum is a sum of eight 10 min measurements recorded in cell #3. The uncertainty of the measurements was determined as the standard deviation of the Poisson distribution, taking into account the background measurements and uncertainty propagation. For all eight measurements, the electron beam energy *E*_e_ was 14.0 GeV, the energy of emitted photons was 9.6 keV, and the undulator gap was 14.0 mm. The number of electron bunches, and hence the charge rate *Q*/*t*, was also constant.

Measurements and simulations are in good agreement. This means that the signal registered by the spectrometer originates from electrons hitting the beam pipe at the point where its aperture changes from elliptical to cylindrical, before the absorber. Measurements presented in Fig. 3 ▸ are representative of the measurements taken in cell #3, as the shape of the energy distribution was the same during constant lasing. The main part of the presented spectrum, with the maximum signal measured around 100 keV, comes from bremsstrahlung photons. It is the result of an electromagnetic cascade initiated by electrons hitting the beam pipe wall. They may either be a part of an electron halo or a contribution of a dark current coming from upstream of the undulator system. Due to the high electron energies involved, emitted bremsstrahlung radiation is sufficiently energetic to produce electron–positron pairs, resulting in the emission of 511 keV photons. These photons are registered by the spectrometer and also reproduced by *Geant4* simulations. A small energy shift between measurements and simulations is believed to be caused by the uncertainty in the energy calibration of the spectrometer, which at higher energies can reach up to 1 keV. Comparison with the calibration values provided by the manufacturer showed that the measurements might show a small systematic shift towards higher energies.

**Figure 4:**
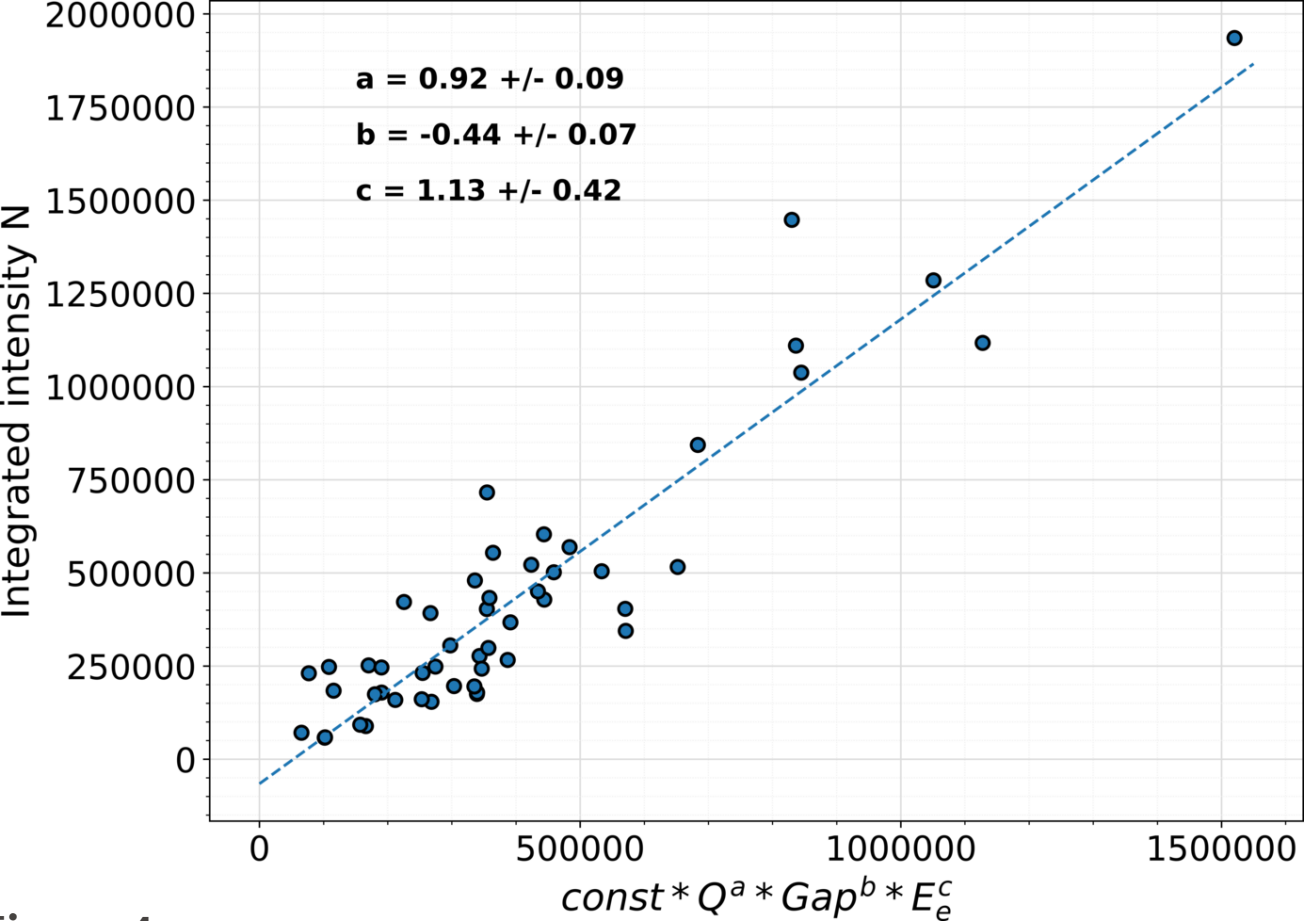
Parametrization of integrated intensity in cell #3.

To investigate how the charge passing through the undulator system, the undulator gap and the electron energy influence the intensity of stray radiation, 10 min gamma-ray spectra were summed for each of the fixed accelerator settings, presented in Table 2 ▸. One way to investigate the dependence of various parameters is through parametrization. In this particular case the charge *Q*, the size of the undulator gap and the electron energy *E*_e_ are likely to be impacting the intensity of the radiation measured by the detector. Hence, the integrated intensity *N* between 30 keV and 1500 keV were then parametrized using the formula
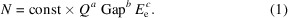
Parameters *a*, *b* and *c* were obtained by a linear regression,
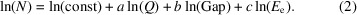
Fig. 4 ▸ shows the result of the fit to the integrated intensity, with fit parameters shown in Table 3 ▸. Ideally, the slope should be equal to one and any deviation may be caused by an insufficient set of parameters or by the precision of the measurements. The intensity of the measured signal increases linearly with the charge. There is no way of telling if this relation is caused by halo electrons deviated from the main beam or by dark current originating in the linear accelerator modules, as they are measured together with the main beam as the total charge passing through the undulator. The simulated electron halo appears to be sufficient to model the measured signal, but the role of the dark current is intriguing and should be addressed in the future.

The electron energy dependence is almost linear, but the uncertainty is large. When the electrons travel through the aluminium vacuum vessel wall, they slow down emitting bremsstrahlung radiation. Both intensity and energy of this radiation depends both on the material and on the electron energy. The data in Fig. 3 ▸ are the convolution of the bremsstrahlung spectrum that passes through the vacuum vessel wall and the response of the detector.

On the other hand, the measured signal is higher for the smaller undulator gap. For a smaller undulator gap, the magnetic field at the beam axis, and hence the undulator parameter *K*, is higher. The amplitude of the electrons’ sinusoidal trajectory, and hence the amplitude of the electron halo, increases for higher *K* values, which means that the electrons close to the beam pipe wall are more likely to hit the beam pipe and create stray radiation.

The measurements shown in Fig. 4 ▸ indicate that either lower electron beam energy or higher undulator gap will produce less stray radiation than higher electron beam energy or smaller undulator gap. It means that the intensity of the stray radiation can be minimized by careful choice of accelerator settings. For the total beam charge of 5.3 × 10^−3^ C passing through SASE1, the integrated counts for measured spectra shown in Fig. 3 ▸ was approximately 2 × 10^5^. The integrated intensity of the simulated spectrum shown in Fig. 3 ▸ was approximately 3 × 10^4^ for 1.6 × 10^6^ electrons. Taking into account that measured intensity depends linearly on the charge, comparison between measurements and simulations shows that the ratio between electrons hitting the beam pipe to all electrons passing through the undulator system is approximately 10^−10^. This is in agreement with previous estimations (Falowska-Pietrzak *et al.*, 2023 ▸). In addition, RADFETs placed in cell #3 do not show any dose increase during regular user operation. This means that, although the spectrometer registers stray radiation, its intensity in the upstream part of the undulator system is too low to pose a risk at current operation to either permanent magnets or diagnostic equipment.

### 3.2. Downstream part of SASE1 – cell #19

**Figure 5:**
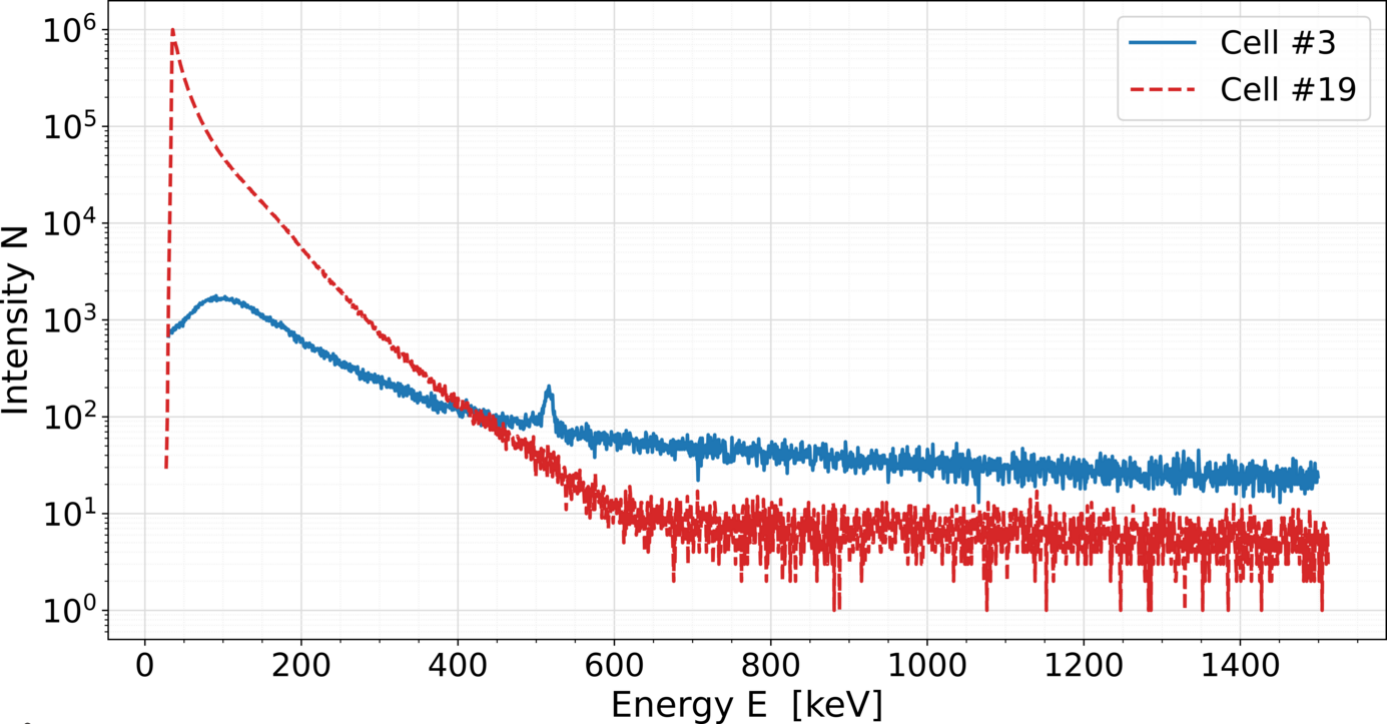
Comparison between gamma-ray spectra measured in cell #3 and cell #19 during the same time.

Fig. 5 ▸ shows a comparison between measurements recorded in cell #3 and cell #19 during the same time and for fixed machine settings (electron energy of 14.0 GeV, undulator gap of 14.0 mm, photon energy of 9.6 keV, constant charge rate). The intensity of the signal measured in cell #19 is always higher compared with cell #3 for energies lower than approximately 400 keV. The higher stray radiation field in cell #19 in the lower energy range is supported by unshielded RADFET measurements. This shows that the stray radiation field present in the downstream undulator cells is dominated by low-energy synchrotron radiation associated with the SASE process, registered by both unshielded RADFET and spectrometer.

The CZT spectrometer suffered from significant dead-time losses due to high-intensity pulsed radiation. Therefore, a quantitative comparison between gamma-ray spectra measured in cell #19 during different times (and hence for different machine settings) was not possible. However, during regular user operation and continuous lasing, the unshielded RADFETs in the downstream undulator cells show a steady increase in absorbed dose (Wolff-Fabris *et al.*, 2019 ▸). Therefore, the impact of different machine settings on the intensity of the stray radiation field in the downstream undulator cell could be investigated with RADFETs.

**Figure 6:**
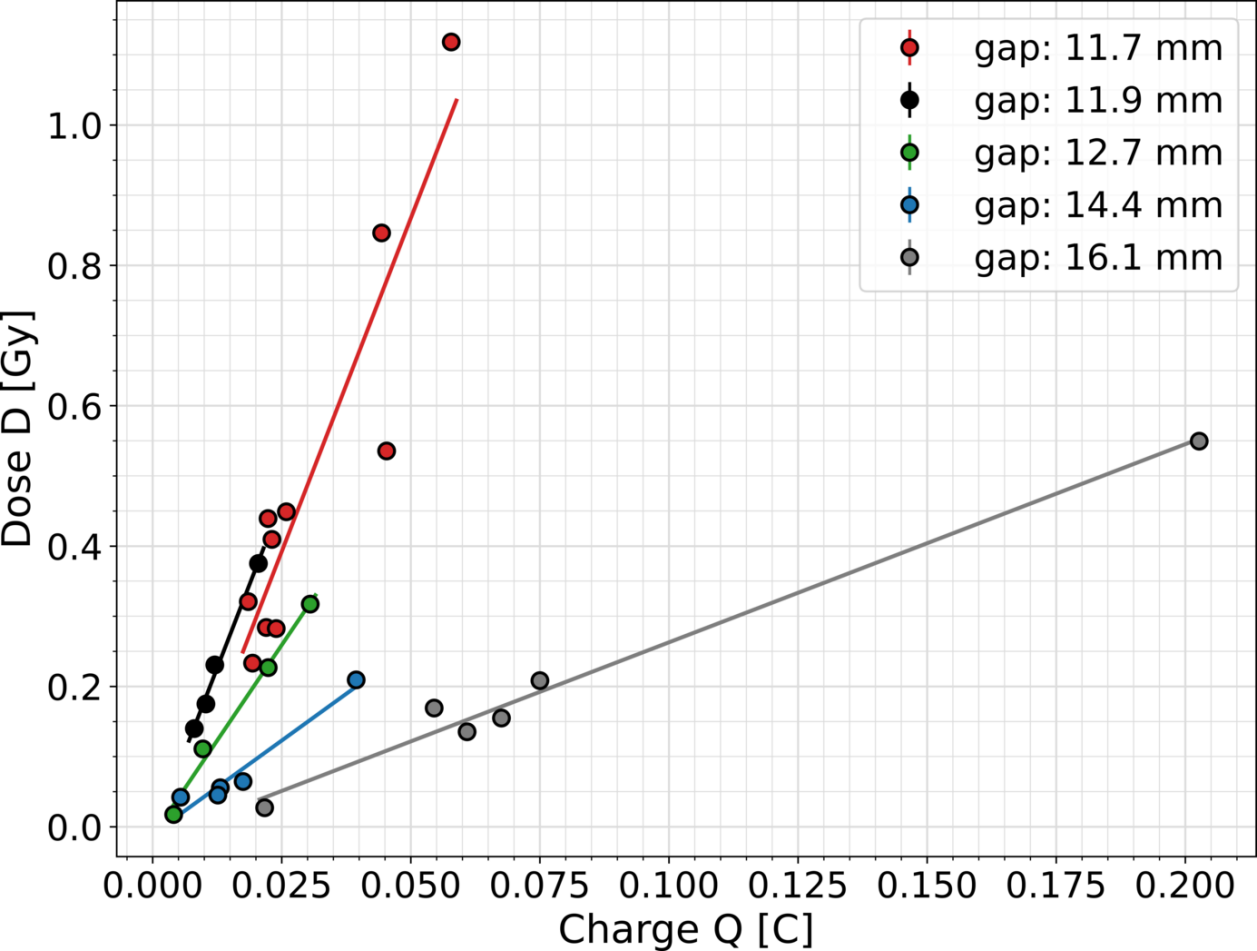
Absorbed dose increase to the unshielded RADFET as a function of charge passing through SASE1 for an electron beam energy of 16.3 GeV and different undulator gaps.

The absorbed dose as a function of charge passing through the SASE1 undulator system is shown in Fig. 6 ▸ for different undulator gaps set according to the FEL delivery to user operations. In all cases, the electron beam energy was 16.3 GeV. The dose measured by the RADFET increases linearly with the charge passing through the undulator system.

**Figure 7:**
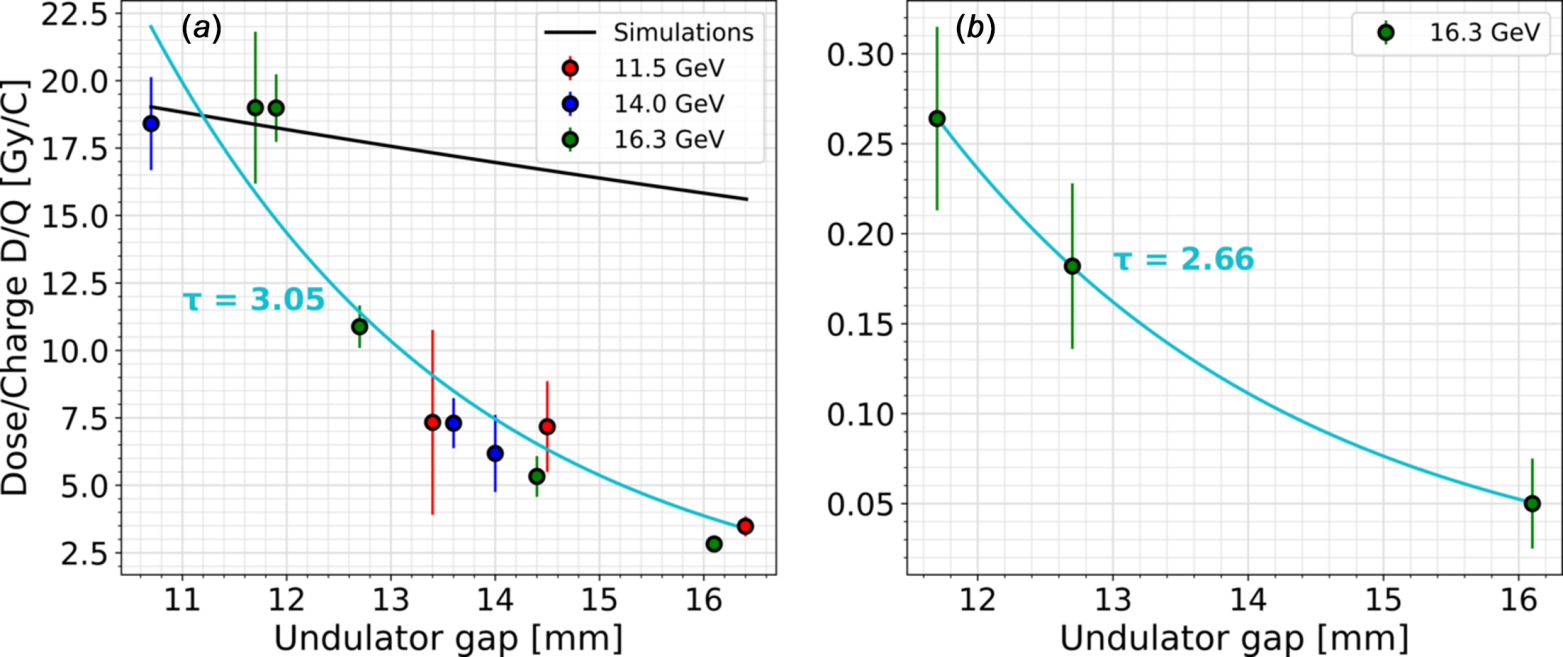
Dose per charge ratio dependence on the undulator gap for different electron beam energies. (*a*) Unshielded RADFET. (*b*) Shielded RADFET. The black line shows the simulations performed previously (Falowska-Pietrzak *et al.*, 2023 ▸), normalized to the dose per charge ratio measurements. The blue line shows an exponential function fitted to measurements, with exponential decay τ.

Absorbed dose per charge ratios were determined from linear regressions shown in Fig. 6 ▸. This was also done for the electron energies of 11.5 GeV and 14.0 GeV. Dose per charge as a function of undulator gap for different electron energies is shown in Fig. 7 ▸(*a*). Regardless of the electron energy, the dose per charge decreases exponentially with increasing undulator gap [the blue line in Fig. 7 ▸(*a*)] and ranges from nearly 20 Gy C^−1^ for the undulator gap of 10.7 mm to around 3 Gy C^−1^ for almost 16.5 mm. These measurements are consistent with the dose per charge ratios measured in cell #17 for an electron energy of 14.0 GeV and undulator gaps of 10.7 mm and 13.7 mm at the early stage of EuXFEL user operation (Wolff-Fabris *et al.*, 2019 ▸).

As previously mentioned, the RADFET’s position is not fixed relative to the beam pipe, as it is mounted at the entrance of the undulator segment with a movable undulator gap. This was taken into account by comparing the measurements with previous simulations as shown in Fig. 7 ▸(*a*). Simulations and Gafchromic film measurements show an exponential decrease of the dose in the vertical direction from the beam pipe (Falowska-Pietrzak *et al.*, 2023 ▸). However, over the short gap range, the simulated dose decrease appears to be linear [black line in Fig. 7 ▸(*a*)].

The exponential function was fitted to the measured dose per charge ratios, according to the formula
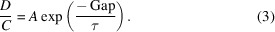
The exponential decay τ of the fitted curve visible in Fig. 7 ▸(*a*) is 3.05. The measured dose per charge ratios show more rapid decrease with increasing undulator gap than the previous simulations. This means that factors other than the distance from the RADFET to the beam pipe have an impact on the stray radiation measured in downstream undulator cells. The simulated dose decrease shown in Fig. 7 ▸(*a*) is a result of electrons interacting with the beam pipe wall at different points along the undulator segment and intersection. The comparison with measurements shows that the radiation field in the downstream part of the undulator segment does not originate from electron losses.

The dose per charge ratio, measured also in cell #19 but with the shielded RADFET, is shown in Fig. 7 ▸(*b*). The measurements also follow an exponential decrease, with a decay τ of 2.66. The dose per charge ratio measured for the shielded RADFET is approximately 1–2% of the signal obtained with the unshielded RADFET, regardless of the undulator gap. This means that the intensity of high-energy radiation in cell #19 is marginal compared with the low-energy synchrotron background dominating the radiation field.

## 4. Conclusions

In this work, the gamma energy distribution of the stray radiation close to the beam pipe in the upstream and downstream part of the SASE1 undulator system was investigated. In addition, the impact of charge passing through the undulator system, undulator gap and electron energy on radiation intensity was analysed based on gamma spectrometers and RADFET dosimeters measurements. Gamma-ray measurements in cell #3, supported by *Geant4* simulations, confirm that the stray radiation field in the upstream part of the SASE1 undulator system originates from GeV electrons striking the beam pipe at points where its aperture changes. Parametrization of the signal registered in cell #3 shows that the intensity is approximately linear to the charge and electron energy. It increases with decreasing undulator gap, which can be explained by more electrons impacting the beam pipe wall when horizontal trajectory increases. Parametrization of the radiation intensity also shows that stray radiation levels can be minimized if accelerator settings are chosen carefully.

The intensity of the signal registered in cell #19 during user operation is several orders of magnitude higher than in the upstream part of SASE1. Because of the high intensity of low-energy synchrotron photons, the dead-time losses in the gamma-ray spectrometer impact the energy spectra measurements. For possible future measurements in the downstream undulator cells, the detector should be placed further away from the beam pipe to limit the intensity of the incoming radiation. The absorbed dose in cell #19 increases with the number of electrons passing through the undulator system. Similarly, as in cell #3, for fixed electron beam energy and electron charge the stray radiation intensity decreases with larger undulator gap. RADFET measurements show that the radiation field in cell #19 is dominated by low-energy synchrotron radiation. Comparison between simulations and measurements confirm that the electron interactions with the beam pipe are not responsible for doses measured in the downstream undulator cells.

## Figures and Tables

**Table 1:** Summary of Monte Carlo simulation parameters (Sechopoulos *et al.*, 2018 ▸)

Item name	Description
Code, version	*Geant4*, version 11.0.
Geometry	Two undulator segments separated by an intersection (see Fig. 2 ▸)
Source	14.1 GeV electrons (*G4GeneralParticleSource)*, ring shape
Physics	Physics reference list FTFP_BERT_HP
Scoring	Energy deposited in the volume
No. of simulated events	1.6 × 10^6^ primary particles

**Table 2:** Different accelerator settings for which the integrated intensity *N* was measured

Electron energy *E*_e_ (Gev)	Undulator gap (mm)	Charge *Q* (C × 10^−3^)
10.6	15.9	2.6, 7.9, 9.3, 9.5, 10.5, 10.6, 31.3
10.6	16.5	5.6
11.5	13.4	7.4, 7.6, 10.0, 12.3
11.5	14.5	7.8, 10.0, 21.2
11.5	14.7	2.4, 4.2, 6.2, 28.9
11.5	15.0	10.7
11.5	16.4	1.6, 4.2, 8.7, 10.8, 11.5, 14.6, 16.8
14.0	14.0	4.7, 5.3, 6.0, 6.0, 10.6
14.0	62.0	3.3, 3.4, 5.4, 5.7, 9.0, 9.0
14.0	210.0	3.7, 10.5, 11.9, 17.1, 24.1
16.3	11.9	4.9, 6.8, 9.9, 23.7
16.3	13.1	3.1, 5.2, 12.8, 13.0

**Table 3:** Parametrization fit parameters

Variable	Function parameter	Value
Charge, *Q* (C)	*a*	0.92 ± 0.09
Undulator gap (mm)	*b*	−0.44 ± 0.07
Electron energy, *E*_e_ (GeV)	*c*	1.13 ± 0.42

## Data Availability

Data will be made available on request
